# A Vital Question for Some Voluntary Hospitals

**Published:** 1917-08-25

**Authors:** 


### A VITAL QUESTION FOR SOME VOLUNTARY HOSPITALS.

LAST week we published a report of a speech made by the Chairman of the Board of Management of King Edward VII.'s Hospital, Cardiff. Colonel Bruce Vaughan quoted Sir Garrod Thomas to the effect that many hospitals were largely staffed with unqualified men, whilst Major David Davies had directed the Premier's attention to the necessity of inquiring into the relative claims of the R.A.M.C. and civilian doctors. Colonel Vaughan declared "the resident staff in the Cardiff Hospital had not been so efficient as it should be. The present difficult and haphazard method of staffing the important voluntary hospitals in the kingdom was a scandal and ought to be inquired into, and a remedy found without delay." Colonel Vaughan suggested that as far as the resident staffs of the great hospitals of the country were concerned, the War Office should delegate a sufficient number of newly qualified men to act as residents, each for a fixed period. This hospital training would do much to fit them for their duties in the field, and would at the same time add greatly to the efficiency of the general hospitals.

There is nothing new in the suggestion to the War Office to delegate a sufficient number of newly qualified men to act as residents for six months. The plan has been tried and proved very successful in some metropolitan hospitals and elsewhere. It has not been consistently pursued or extended to the majority of hospitals, partly, we understand, from a shortage of men, and partly from the demand everywhere for qualified practitioners, so that directly the student receives his tin case and diploma he is ambitious to go out on his own account to practise, a course which is not good for his future if he is ever to be a competent practitioner or just to his patients; nor does it fulfil the present needs of the nation. We believe attempts have been made to control the tin-case practitioner and utilise him in the way Colonel Vaughan suggests, but with indifferent success. Anyway, there is undoubtedly a very urgent demand for reform, and the efficient provision of an adequate number of qualified men to take resident posts. We have reason to hope that the profession in the United States may be able to send over a considerable number of qualified practitioners to help the Mother Country in the present war emergency. We hope the War Office will reconsider the whole position, and to take steps to co-operate with hospital managers who, like Colonel Vaughan, are untiring in their devotion to the best interests of the civil and military patients.

There is another and a very serious side to the questions involved by Colonel Vaughan's statement that "the resident staff at Cardiff and elsewhere has not been so efficient as it should be." This lack of efficiency within the hospitals could not constitute a danger or be injurious to the interests of the patients, if the medical staff of every hospital were to co-operate and to enable each institution to make adequate provision, whereby the maximum of efficiency in treatment could be guaranteed. This step would not be difficult where arrangements are made with a competent physician and a competent surgeon to supervise the work entrusted to the residents, in connection with the medical and surgical cases, and the casualty department, in every hospital during the war. It is unthinkable that minor or other operations should be suffered to be performed by residents, whose incompetence may be demonstrable by the after-effects in the case of the patients, and especially children.

Such things should not occur, and the hospital world owes thanks to Colonel Vaughan for raising the question. We hope that without a moment's avoidable delay every hospital committee will look into this matter, and call a conference with the members of the honorary medical staff within reach. Then everywhere, the necessary steps can be taken, to render it impossible that the patients of any voluntary hospital shall be permitted to be solely dependent on members of a resident staff who have not proved so efficient as they should be.

